# Bispectrum Estimation of Electroencephalogram Signals During Meditation

**Published:** 2012

**Authors:** Ateke Goshvarpour, Atefeh Goshvarpour, Saeed Rahati, Vahid Saadatian

**Affiliations:** 1 Department of Biomedical Engineering, Islamic Azad University, Mashhad Branch, Iran.; 2 Department of Electrical Engineering, Islamic Azad University, Mashhad Branch, Iran.; 3 Department of Psychiatry, Islamic Azad University, Mashhad Branch, Faculty of Medicine, Iran** .**

**Keywords:** Bispectrum Estimation, Electroencephalogram, Meditation, Phase Coupling

## Abstract

**Objective:** Electroencephalogram is a reliable reflection of many physiological factors modulating the brain. The Bispectrum is very useful for analyzing non-Gaussian signals such as EEG, and detecting the quadratic phase coupling between distinct frequency components in EEG signals.The main aim of this study was to test the existence of nonlinear phase coupling within the EEG signals in a certain psycho-physiological state; meditation.

**Methods:** Eleven meditators and four non-meditators were asked to do meditation by listening to the guidance of the master, and 10 subjects were asked to do meditation by themselves. Bispectrum estimation was applied to analyze EEG signals, before and during meditation. EEG signals were recorded using 16-channel PowerLab. ANOVA test was used to establish significant changes in Bispectrum parameters, during two different states (before and during meditation).

**Results:** Mean Bispectrum magnitude of each channel increased during meditation. These increments of phase coupling are more obvious in occipital region (Pz channel) than frontal and central regions (Fz and Cz channels). Besides that phase coupled harmonics are shifted to the higher frequencies during meditation.

**Conclusion:** Bispectrum methods can be useful for distinction between two states (before and during meditation).

## Introduction

Meditation, a technique that frees the mind from distractions and allows for communication with the Master Within, can lead to numerous physical, mental and spiritual benefits. Meditation is a unique state of consciousness with associated changes in the physiological and psychological functions in the brain. Physiological research has shown that meditation characterized by marked reductions in metabolic activity, increased orderliness and integration of brain functioning, decreased peripheral vascular resistance, and increased cerebral blood flow(1).

Electroencephalogram (EEG) is a recording of spontaneous brain electrical activity by means of electrodes located on the scalp. It is playing a more and more important part in analyzing brain activities and diagnosing brain diseases now, so it is one of the most important neurological signals and is often used to detect brain’s neurological dysfunction and neurophysiological disorders.

Currently, most existing schemes for extracting EEG features are based on autoregressive (AR) models or adaptive AR models (AAR)(2, 3) and power spectral density (PSD)(4).In practice, meaningful EEG features can be extracted from various frequency bands of recorded EEG signals(5-7).

However, conventional methods for feature extraction based on AR models and PSD assume linearity, Gaussianality and minimum-phase within EEG signals, i.e., the amplitudes of EEG signals are normally distributed, their statistical properties do not vary over time, and their frequency components are uncorrelated(8). Under these assumptions, the EEG signal is considered as a linear superimposition of statistically independent sinusoidal or other wave components, and only frequency and power estimates are considered while phase information is generally ignored. In reality, EEG signals are generated by a typical nonlinear system. Thus EEG signals would have many sinusoidal components of distinct frequencies, interacting nonlinearly to produce one or more sinusoidal components at sum and difference frequencies, which cannot be completely characterized by auto-correlation functions, as done by AR models or PSD estimation methods.

To overcome this limitation, this paper proposes a set of features for EEG, which includes higher order analysis based on the Bispectrum of EEG signals.

Bispectrumanalysis has several additional characteristics that may be advantageous for processing EEG signals: Gaussian sources of noise are suppressed, thus enhancing the signal-to noise ratio for the non-Gaussian EEG, and Bispectrumanalysis can identify nonlinearities, which may be important in the signal generation process.

Since higher order spectrum methods do not require the knowledge of thenon-Gaussian distribution, they become more attractive in practical application problems of signal processing because the non-Gaussian distribution of the data is usually unknown or difficult to estimate.Several authors used the Bispectral analysis for investigating the EEG. Bullock *et al.*(9)used Bispectral analysis for the detection of short-term non-stationarity and nonlinearity. Muthuswamy*et al.*(10)detected phase coupling between two frequency components within the delta-theta frequency band of EEG bursts. Gajraj*et al*. (11)applied the EEG Bispectrum in order to distinguish the transition from unconsciousness to consciousness. Shils*et al*. (12)studied the interactions between the electrocerebral activities resulting from stimulation of the left and right visual fields. They observed nonlinear interactions between visual fields by means of Bispectral analysis. Schack et al. **Error! Reference source not found.** (13) examined the existence of nonlinear phase coupling between theta and gamma EEG activities within the frontalarea during memory processing.

The aim of the present study was to evaluate the nonlinearity of EEG signals. For this purpose, we used the EEG signals (Fz, Cz and Pz channels) of two states (before meditation and during meditation). Bispectrum of each channel was estimated via two techniques: parametric and the direct (FFT) method. 

## Materials and Methods

*Data Selection*

This study involved the EEG signals of 25 healthy women.The number of meditators was selected according to their level of meditation training; all healthy advanced individuals referred to the meditation clinic were asked to participate in this study. Accessibility, be healthy and the amount of meditation training were the most significant factors in our data collection. Fifteen subjects: 11 meditators (mean meditation experience of 5 to 7 years) and 4 non-meditators were asked to do meditation by listening to the guidance of the master. The other ten subjects were asked to do meditation by themselves. They were considered to be at an advanced level of meditation training (mean meditation experience of 7 years).All subjects were in good general health and did not follow any specific heart diseases or psychological disorders. Informed written consent was obtained from each subject after the experimental procedures were explained.

The experimental procedure was divided into two different stages: Subjects were first instructed to sit quietly for 5 minutes and kept their eyes closed. After that, they performed meditation. Meditation prescribes a certain bodily posture. They sit on a cushion 5 to 10 centimeters thick that is placed on blanket. They cross their legs so that one foot rests on the opposite thigh with the sole of their foot turned up and with their knees touching the blanket (lotus or half-lotus position). The torso should be kept straight, but it should not be strained. The head should be kept high with eyes closed. During this session, the subjects sat quietly and focusing on the breath. 

The meditation EEG signals were recorded in meditation clinic using 16-channel PowerLab (ADInstruments, Australia) system. EEG activity was recorded using three electrodes (Fz, Cz and Pz channels) according to the International 10–20 System, referenced to linked ear lobe electrodes. A digital notch filter was applied to the data at 50Hz to remove any artifacts caused by alternating current line noise. The sampling rate was 400Hz.

*Bispectrum estimation*

The Bispectrum quantifies the relationship among the underlying sinusoidal components of the EEG. Specifically, Bispectrumanalysis examines the relationship between the sinusoids at two primary frequencies, f_1_ and f_2_, and a modulation component at the frequency (f_1_ + f_2_). This set of three frequency components is known as a triplet (f_1_, f_2_, and f_1_ + f_2_). For each triplet, the Bispectrum, B (f_1_, f_2_), a quantity incorporating both phase and power information, can be calculated as described below (14).Strong phase coupling implies that the sinusoidal components at f_1_ and f_2_ may have a common generator, or that the neural circuitry they drive may, although some nonlinear interaction, synthesize a newdependent component at the modulation frequency, f_1_ + f_2_.

$B\left(f_{1},f_{2}\right)=|X\left(f_{1}\right)\mathrm{.X}\left(f_{2}\right).X^{*}\left(f_{1},f_{2}\right)|$              (1)

Where B(f_1_,f_2_) is the Bispectrum at frequencies f_l_ and f_2_, X(f) and X(f) are discrete Fourier transform coefficients at frequencies f_l_ and f_2_ respectively. X^*^ is the complex conjugate of X. This multiplication is the heart of the Bispectrum determination: if at each frequency in the triplet, there is a large spectral amplitude (i.e., a sinusoid exists for that frequency) and if the phase angles for each are aligned, then the resulting product will be large; if one of the component sinusoids is small or absent or if the phase angles are not aligned, the product will be small. Finally, the complex Bispectrum is converted to a real number by computing the magnitude of the complex product.

To ensure statistical accuracy a long signal length is divided into P epochs and the Bispectrum of each epoch B_i_(f_1_,f_2_) is formed. The Bispectrum of the total signal is then the averaged value of these bispectra:

Bf1,f2=1p∑Bi(f1,f2)              (2)

- *x(t), t=±1,±2,±3,…* *is a real stationary process that characterized with its' moment.* *We define the expectation,E(.), by* *equation * *‎(3):*

Ext=limn→∞(12N+1)∑x(t)           (3)

- *The first moment is defined by equation* *‎* *(4): *

$\mu =E(x\left(t\right))$           (4)

- *The first moment of x, is called the mean. It is convenient to define the higher order cumulants under the assumption of zero mean,* *µ=0* *. The second moment of x is defined by equation* *‎* *(5):*

$\sigma^{2}\left(t_{1}\right)=E(x(x\left(t\right)x\left(t+t_{1}\right))$            (5)

- *The third moment of x is defined by* *equation* *‎* *(6)* *:*

$\gamma \left(t_{1},t_{2}\right)=E(x(t(x\left(t+t_{1}\right)x\left(t_{1}+t_{2}\right))$           (6)

- *The second moment in* *t*_1_ *=0* *is called variance and the third moment in* *t*_1_ *= t*_2_ *=0* *is called skewness. The skewness is usually defined by* *γ(0,0)/σ* ^3^ *.*

The Fourier transform of second moment is called power spectrum P(f) and the Fourier

$\sigma^{2}\left(t_{1}\right)=\int e^{2\mathrm{\pi i}t_{1}f}P\left(f\right)\mathrm{df}$          (7)

$\sigma^{2}(t_{1},t_{2})=\int e^{2\mathrm{\pi i}{(t}_{1}f_{1}+t_{2}f_{2})}B\left(f_{1},f_{2}\right)df_{1}df_{2}$      (8)

 transform of third moment is called Bispectrum B(f_1_,f_2_).

for t_1_= t_2_=0:

$\sigma^{2}\left(0\right)=\sigma^{2}=\int P\left(f\right)\mathrm{df}$          (9)

$\gamma \left(0,0\right)=\gamma \sigma^{3}=\int B\left(f_{1},f_{2}\right)df_{1}df_{2}$          (10)

σ^2^ is variance and σ^3^ is skewness. Bispectrum can be estimated via several techniques. In this paper we used parametric and the direct (FFT) method. 

In Bispectrum estimation using the direct (FFT-based) method, the data are segmented into possibly overlapping records; the mean is removed from each record, and the FFT computed; the Bispectrum of the k_th_ record is computed as, B_K_(m,n)=X_K_(m).X_K_(n).X_K_^*^(m+n), where X_K_denotes the FFT of the k_th_ record. The Bispectrum estimates are averaged across records.

In Bispectrum estimation using parametric method, AR parameters should be estimated(15).

*Statistical analysis*

In this study, the ANOVA test was used to establish significant changes in Bispectrum parameters, during two different states (before and during meditation).A p value <0.05 was considered significant.

## Results

**Figure 1:**
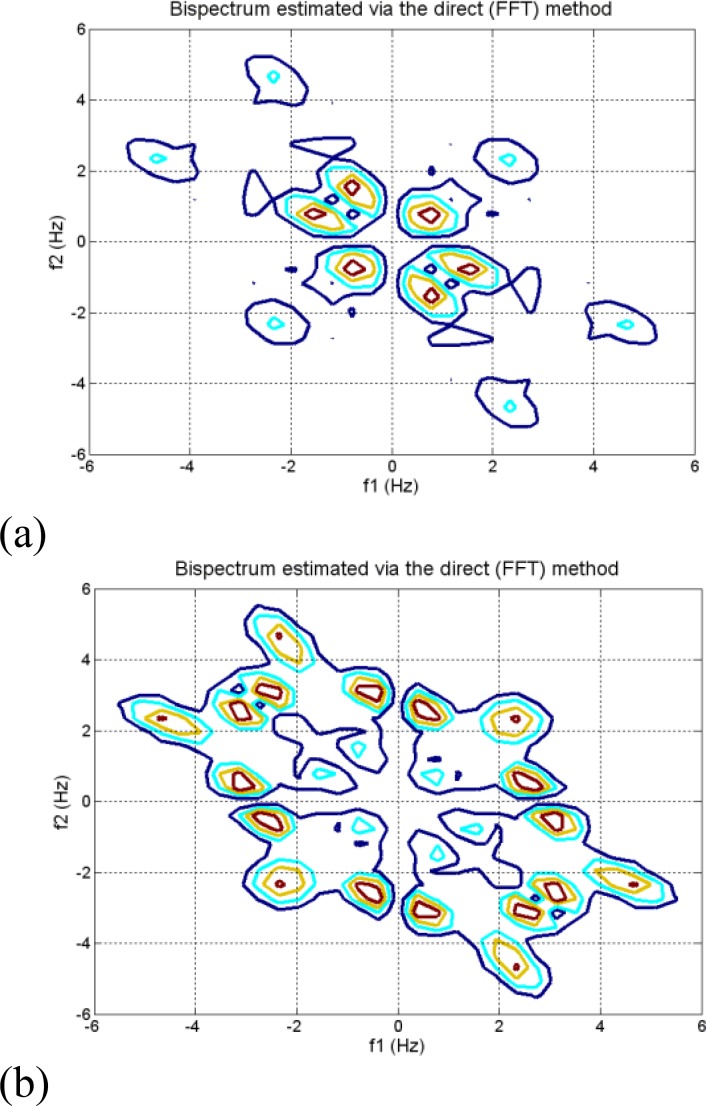
Bispectrum estimation of Pz channel (record 13) via the direct method. (a) Before meditation. (b) During meditation

**Figure2:**
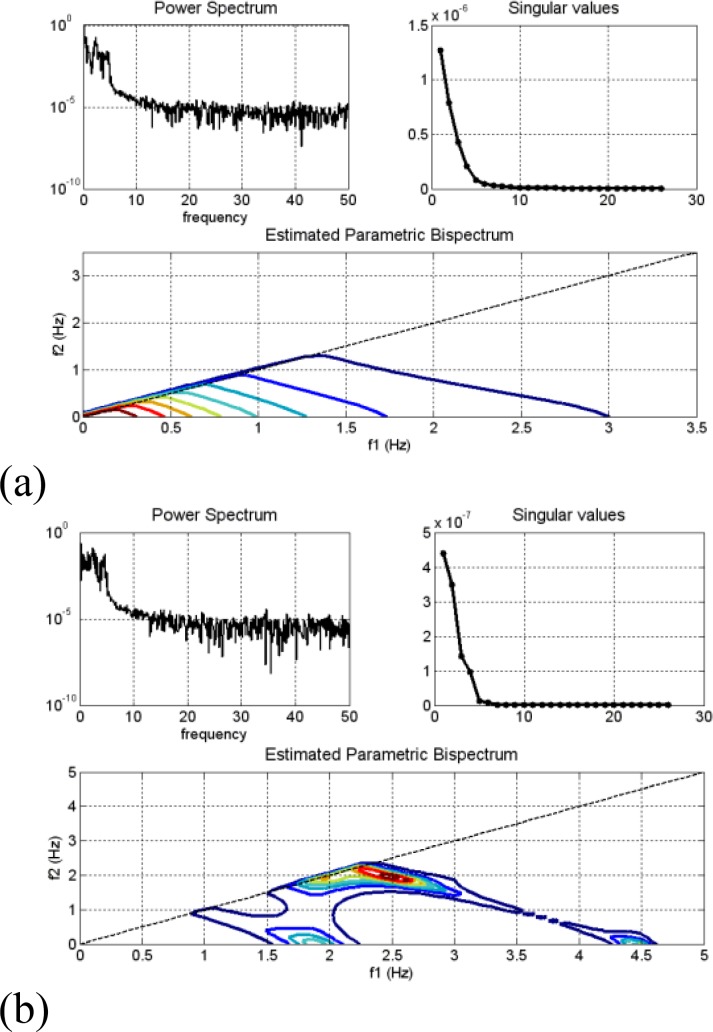
Power spectrum, singular values and estimated parametric bispectrum of Pz channel (record 13). (a) Before meditation. (b) During meditation

Bispectrum of each channel (Fz, Cz and Pz channel) is estimated via two techniques: parametric and the direct (FFT) method using the MATLAB toolbox. In Bispectrum estimation using the direct (FFT-based) method, the FFT length is 512 and the percentage overlap between segments is set to zero. In parametric method, we used an AR order of 10 and the percentage overlap between segments is set to zero. The results of Bispectrum analysis of EEG signals during meditation were compared to before meditation. The Bispectrum estimations of Pz Channel (record 13) are shown in     Figure1  and     Figure2 .

The Bispectrum estimations of EEG signals before and during meditation via the direct method are shown in and the results of parametric method are shown in     Figure2 . The color represents the relative change in amplitude of Bispectrum; red indicating the greatest increase and blue indicating the greatest decrease. According to the phase-coupled harmonics are less than 2.5 Hz before meditation and the phase-coupled harmonics are below 6 Hz during meditation. The Bispectrum shows sharp peaks at around (1,1)Hz (and symmetric locations) before meditation and it shows sharp peaks at around (3,3)Hz , (3,1)Hz and (1,3)Hz (and symmetric locations) during meditation.

Power spectrums of EEG signals (record 13) are shown in left top panel of     Figure2 . Before meditation, the maximum value of the power spectrum is about 10^0^=1but it is about 10^-.056^ during meditation. Therefore, the power spectrum of signal is decreased during meditation.

Singular values of EEG signals (record 13) are shown in right top panel of     Figure2 . Before meditation, there are eight significant singular values (    Figure2 a) and six significant singular values are presented during meditation (    Figure2  b), corresponding to one quadratically coupled triplet. In this study, we used an AR order of 10. As shown in     Figure2 a, the dominant peak is at (f_1_,f_2_)=(0,0), and its magnitude is about 4.12×10^4^. According to     Figure2 b, the dominant peak is at (f_1_,f_2_)=(2.5391,1.9531), and its magnitude is about1.77×10^9^. The dominant peaks indicating the presence of a second harmonic are at 0 Hz before meditation and 4.49 Hz during meditation. According to     Figure2  the phase-coupled harmonics are less than 3 Hz before meditation and the phase-coupled harmonics are below 4.5Hz during meditation.

**Figure 3:**
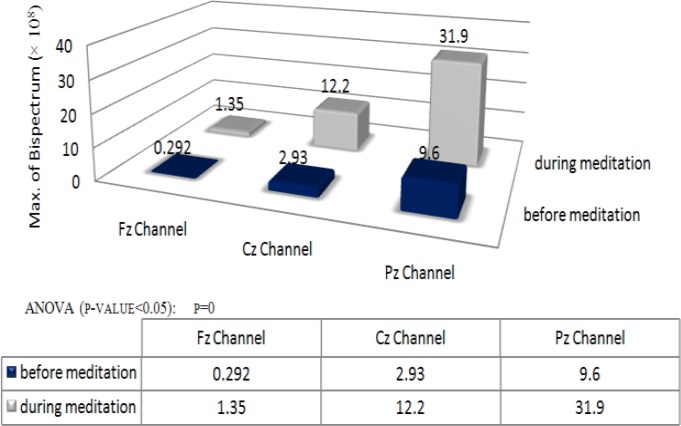
Average value of maximum magnitudes of Bispectrum estimation via parametric method before and during meditation.

Average value of maximum magnitude of Bispectrum estimation via parametric method is shown in     Figure 3 . 

Mean Bispectrum magnitude seems to increase from before meditation to during meditation. These increments of phase coupling are more obvious in occipital region (Pz channel) than frontal and central region (Fz and Cz channels).Furthermore, thesmall value of p (p<0.05) suggests that at least one sample mean is significantly different than the other sample means.

## Discussion

Some previous works showed that meditation was accompanied by theta and alpha synchronization(5). A recent study (7) reported that slow alpha power (alpha1) and fast theta power (theta 2) on EEG increased predominantly in the frontal area during meditation.

There are situations in practice where because of interaction between two harmonic components of a process there is contribution to the power at their sum and/or difference frequencies. Such a phenomenon which could be due to quadratic nonlinearities gives rise to certain phase relations called quadratic phase coupling. In certain applications it is necessary to find out if peaks at harmonically related positions in the power spectrum are, in fact, coupled. Since the power spectrum suppresses all phase relations, it cannot provide the answer. The Bispectrum, however, is capable of detecting and quantifying phase coupling. Therefore, the Bispectrum is very useful for analyzing non-Gaussian signal such as EEG, and detecting the quadratic phase coupling between distinct frequency components in EEG signal.Thus, it reflects the interaction of neuronal subcomponents exhibiting oscillations at different frequencies. The main goal of this study was to examine the phase coupling of EEG signals in the specific psychophysiological state. For this purpose we estimated the Bispectrumof EEG signals (Fz, Cz and Pz channels) of twenty fivehealthy women.

The results show that the maximum value of the power spectrum is increased during meditation (    Figure 3 ). The mean Bispectrum magnitude of each channel is increased during meditation(p<0.05). These increments of phase coupling are more obvious in occipital region (Pz channel) than frontal and central regions (Fz and Cz channels). Besides that phase-coupled harmonics are shifted to the higher frequencies during meditation. 

It is known that EEG signals are generated from human brain which is a system with highly nonlinear dynamics, but there is no evidence indicating that the activation of human brain is Gaussian. We believe that more of the information available in the EEG signals must be exploited, such as the information of nonlinearity and non-Gaussianality. The key advantages of the proposed feature extraction method lie in that: (1) the proposed feature set contains high-order statistics information, while the widely used conventional features with only second-order measures (such as the power spectrum and autocorrelation functions) do not. As a consequence, non-minimum-phase signals, such as EEG signals, cannot be correctly characterized by the second-order measures. Moreover, some types of phase coupling in EEG signals which is associated with nonlinearities cannot be correctly identified by the second-order measures (2) . The proposed feature set is less affected by Gaussian background noise than the conventional features with only second-order measures due to the property of Bispectrum: the Bispectrum of Gaussian signal is zero.

The results indicate that the Bispectral analysis of the EEG can reveal extra information non obtainable from the power spectrum and may provide insights regarding the formation of EEG signals within different brain structures during two states (before and during meditation).

There is plenty of room for research in this area with several lines of investigation to be pursued. For example, one could study the use of trispectrum estimation and the estimatingof fourth-order cumulants via AR and ARMA models for phase estimation. This approach will turn out to be very useful in the case of non-Gaussian processes like EEG signals. One could also investigate the use of nonlinear features for EEG analyzing during psycho-physiological states.Similar study on more subjects is necessary to establish such trend.

## Authors' contributions

The work presented here was carried out in collaboration between all authors. The co-authors provided some general ideas that initialized the work. AG and AG conceived and designed the evaluation, collected experimental data, performed the analysis, interpreted the results, and drafted the manuscript.Valuable guidance and critical revision of the article are credited toSR.VS contributed to the design of the experimental data protocol and re-evaluated the clinical data.All authors read and approved the final manuscript.

## Notes

**Declaration of interest: **None.
